# Rapidly predicting Kohn–Sham total energy using data-centric AI

**DOI:** 10.1038/s41598-022-18366-7

**Published:** 2022-08-24

**Authors:** Hasan Kurban, Mustafa Kurban, Mehmet M. Dalkilic

**Affiliations:** 1 Applied Data Science Department, San José State University, San Jose, CA 95192 USA; 2 Computer Science Department, Indiana University, Bloomington, IN 47405 US; 3 Department of Electrical and Electronics Engineering, Kırşehir Ahi Evran University, 40100 Kırşehir, Turkey

**Keywords:** Nanoscale materials, Theory and computation

## Abstract

Predicting material properties by solving the Kohn-Sham (KS) equation, which is the basis of modern computational approaches to electronic structures, has provided significant improvements in materials sciences. Despite its contributions, both DFT and DFTB calculations are limited by the number of electrons and atoms that translate into increasingly longer run-times. In this work we introduce a novel, data-centric machine learning framework that is used to rapidly and accurately predicate the KS total energy of anatase ${\mathrm{TiO}}_2$ nanoparticles (NPs) at different temperatures using only a small amount of theoretical data. The proposed framework that we call co-modeling eliminates the need for experimental data and is general enough to be used over any NPs to determine electronic structure and, consequently, more efficiently study physical and chemical properties. We include a web service to demonstrate the effectiveness of our approach.

## Introduction

**Figure 1:**
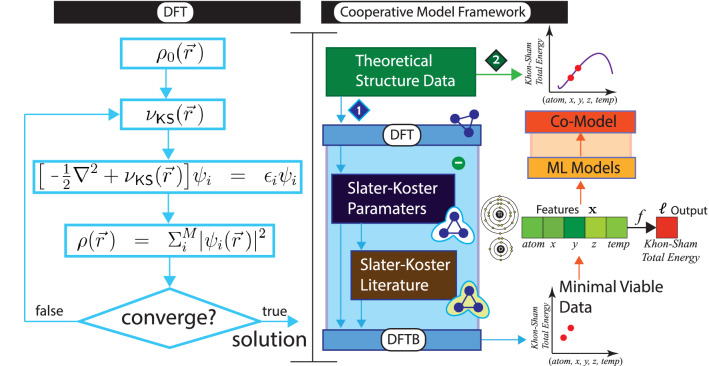
(Left) The DFT algorithm. (Right) The cooperative model framework for ${\mathrm{TiO}}_{2}$ as an example. The first pass $\langle 1 \rangle$ uses DFT/DFTB to produce the *minimal viable data* (the smallest data size that will produce effective co-modeling). Once the co-model is constucted, we can directly submit the atomic geometry data $\langle 2 \rangle$ for ${\mathrm{TiO}}_{2}$ to predict Kohn–Sham Total Energy without having to use DFT/DFTB. Throughout the paper we may abbreviate *Kohn–Sham total energy* to *total energy*. We use typical ML formalism describing inputs as a vector ${\mathbf {x}}$, output as label $\ell$, and classifier as $f:{\mathbf {x}}\rightarrow \ell$.

**Figure 2:**
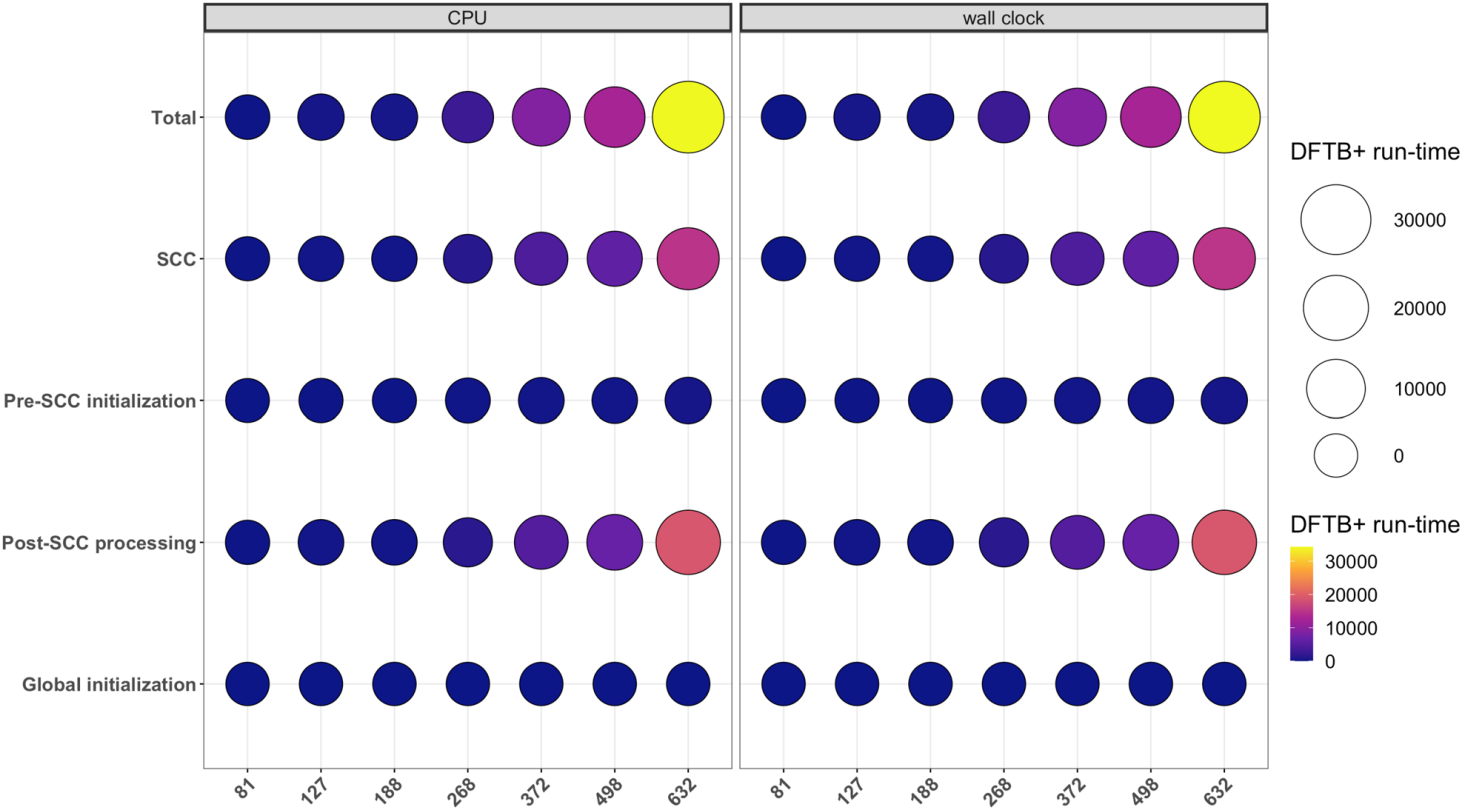
Run-time (seconds) rate of DFTB+ computation as the number of atoms increase. The abscissa show atom numbers and ordinate task names in DFTB+. SCC = diagonalization + density matrix creation, post-SCC processing: eigenvector uniting + energy-density matrix creation + force calculation. The figure is made using *ggplot2, ggpubr, dplyr* and *reshape2* packages in *R*.

Machine Learning (ML) has begun making critical contributions across all the sciences^1–6^. Materials science (MS), a very broad interdisciplinary field that seeks to discover and design new materials, is being particularly impacted^7–11^ from the theoretical^12–16^ to the practical—predicting structural properties^17–21^ and discoverying of new materials *e.g.*, perovskites, nanoparticles, nanoclusters^22–25^. A particularly active area has been using ML to get get new types of interatomic potentials—namely the ML potentials^26–32^ applied to a wide range of material systems^33–37^ with accuracies comparable to those of first-principles calculations. Even specializations within ML itself, such as “class imbalances” (where the significance of data is not reflected in its distribution), offer benefits to MS^38^. The focus of this work is to leverage AI to improve upon the long-standing, traditional quantum modeling technique Density-functional theory (DFT), a *computational* method developed in 1970s, for modelling quantum mechanics that is among the most popular tools of the material scientists [Fig. 1(left)]. DFT became practicable with Kohn–Sham’s formulation (KS)^39^ some 15 years later. KS is the non-interacting Schrödinger equation, which is an iterative procedure minimizing total energy while pairs of electron densities differ. Improving on the computational speed, density-functional tight-biding (DFTB) based on DFT, leverages *experimental* data^40,41^. While there is a considerable growing demand to predict material properties increasing in both size and complexity, fundamental run-time complexity of interacting electrons place hard limits on scaling DFT^42,43^ from polynomial run-time and, hence, DFTB too. Figure 2 illustrates the dramatic bottleneck of run-time as a function of atoms.

Recently, focused use of ML has shown promise to improving DFT^44–46^ in limited ways, but a general framework leveraging ML is still missing, and no work, that we are aware of, is applied to DFTB. To further demonstrate the potential of this novel approach, we apply co-modelling to anatase TiO$_{2}$ NPs used in a wide range of applications^47–50^. Figure 1(right) illustrates our approach with various elements subsequently described in later sections. On the first pass $\langle 1 \rangle$ we visit DFT/DFTB once computing Kohn–Sham total energy for a given temperature and ${\mathrm{TiO}}_2$ nanoparticles (NPs). This is repeated for increasing temperature associated ${\mathrm{TiO}}_2$ NPs creating *minimal viable data*—the smallest data that allows us to build our model. The model itself is an linear ensemble of 147 disparate models selected from a maximum cohort of 230 where either poorly performing correlated models are removed. The collective time to select, train, and test the co-model is orders of magnitude faster than relying on traditional DFT/DFTB. Once the co-model is constructed, the atom locations (geometry) can bypass DFT/DFTB $\langle 2 \rangle$ and be directly sent to the model. To show the effectiveness of this novel approach we have build a web service for ${\mathrm{TiO}}_2$ using this co-model (https://hasan.shinyapps.io/app_1/) that predicts the Kohn–Sham total energy for any temperature in $[0\,{\mathsf {K}},1000\,{\mathsf {K}}]$. Co-modelling is a novel, data-centric, very fast, and effective hybrid of DFT/DFTB and ML. Our approach is supported by a long-standing observation of the bias of using a single model^51^. For our work this means using this approach we can always build more accurate co-models than existing single ML models as our results demonstrate as well.

The remainder of the paper is as follows: “Background and related work” section we provide background on KS and an overview of ML and brief description of the members of ${\mathscr {M}}$. In “Methodology and experimental results” section, we give a detailed description of co-modelling and show its application to the ${\mathrm{TiO}}_{2}$ NPs. “Summary and conclusion” section is the summary can conclusion. Both code and data are publicly accessible. [https://github.com/hasankurban/Kohn-Sham-Total-Energy-Prediction.git].

## Background and related work

### The Kohn–Sham equation

The DFTB formalism contains two major contributions [see Eq. (1)], which are matrix elements (the Hamilton and overlap) and the repulsive potentials^52^. The fundamental idea of the DFTB method is to implement the second order expansion of the Kohn–Sham (KS) DFT where the electronic density $(\rho _0)$ is calculated from a reference function,

1 $$\begin{aligned} E^{total} = E^0[\rho _0] +E^1[\rho _0, \delta \rho ]+ E^2[\rho ,(\delta \rho )^2] \end{aligned}$$

where $\rho _0$ is the sum of neutral atomic densities. The first term of Eq. (1) represents a Kohn–Sham effective Hamiltonian;

2 $$\begin{aligned} E^0[\rho _0] = \hat{H_0} = \langle \eta _{\mu }\ | \ {\hat{H}}[\rho _0]\ | \ \eta _{\nu } \rangle \end{aligned}$$

and the second term is related to the energy due to charge fluctuations

3 $$\begin{aligned} E^1[\rho _0, \delta \rho ] =\frac{1}{2} {\hat{S}}_{\mu \nu } \sum \limits _{X} (\gamma _{AX} + \gamma _{BX})\Delta q_{X} \end{aligned}$$

where ${\hat{S}}_{\mu \nu } = \langle \eta _{\mu }\ |\ \eta _{\nu }\rangle$ is the overlap matrix elements. In the DFTB formalism, the matrix elements of atomic orbitals are pre-calculated and stored. Besides, the second order self-consistent charge (SCC) extension is used in the DFTB method due to the dependence of the DFTB Hamiltonian on the atomic charge. The third term is the repulsive potential which is approximated as a sum of two-center repulsions,

4 $$\begin{aligned} E^{2}[\rho , (\delta \rho )^{2}] =\frac{1}{2}\sum \limits _{A,B}V_{AB}(|\vec {R}_{A}-\vec {R}_{B}|) \end{aligned}$$

with pair potentials based on the respective atom types and the interatomic distance $R_{AB} = \vec {R}_A -\vec {R}_B.$ Typically this problem is solved by a self-consistent approach. Schematic representation of the self-consistency cycle in Kohn–Sham equations is given in Fig. 1(left).

### The method of calculations

Temperature dependent structures of anatase phase TiO_2_ NPs have been obtained using molecular dynamics (MD) methods implemented in DFTB+ code^53^ with the hyb-0–2^54,55^ set of Slater Koster parameters. Thermal equilibrium is controlled by the NVT ensemble during whole simulations. The time step of MD was chosen as 1 fs. The temperature of the NPs was increased by $50\,{\mathsf {K}}$ up to $1000\,{\mathsf {K}}$.

### Machine learning

#### Related work

Ellis et al.^56^ introduces an ML based framework, where the feed-forward neural network is used, to speed up DFT calculations over aluminum at ambient density $(2.699\, {\mathsf {g}}/{\mathsf {cc}})$ and temperatures up to the melting point $(933\, {\mathsf {K}})$. The authors show that their model can accurately predict solid and liquid properties of aluminum. Li et al.^57^ shows the relations between density and energy can be iteratively and accurately learned with very small data and ML models, which have better generalizability, by including KS equations into the training data. KS equations are solved while learning exchange-correlation functional with neural networks which improve generalization. Chandraskeran et al.^58^ demonstrates ML models can predict the electronic charge density and DOS from atomic configuration information. Instead of learning of a specific representation of the local electronic properties, the authors propose a grid-based learning and predictions scheme which is more memory intensive, but can routinely handle large systems and improve run-times. Brockherde et al.^59^ perform the first MD simulation with a machine-learned density functional on malonaldehyde constructing more accurate density functionals for realistic molecular systems. Schleder et al.^60^ gives an overview of ML techniques used in modern computational materials science to design novel materials and explain the present challenges and research problems.

## The cooperative model framework (co-model)

We first give an informal description of co-modelling to prepare for the detailed description for Algo. 1.
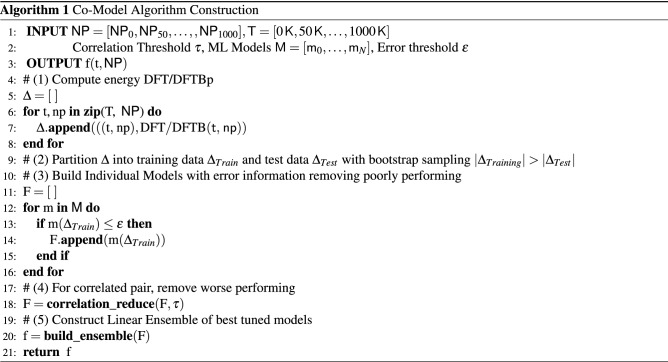


### An overview of co-modelling

**Figure 3:**
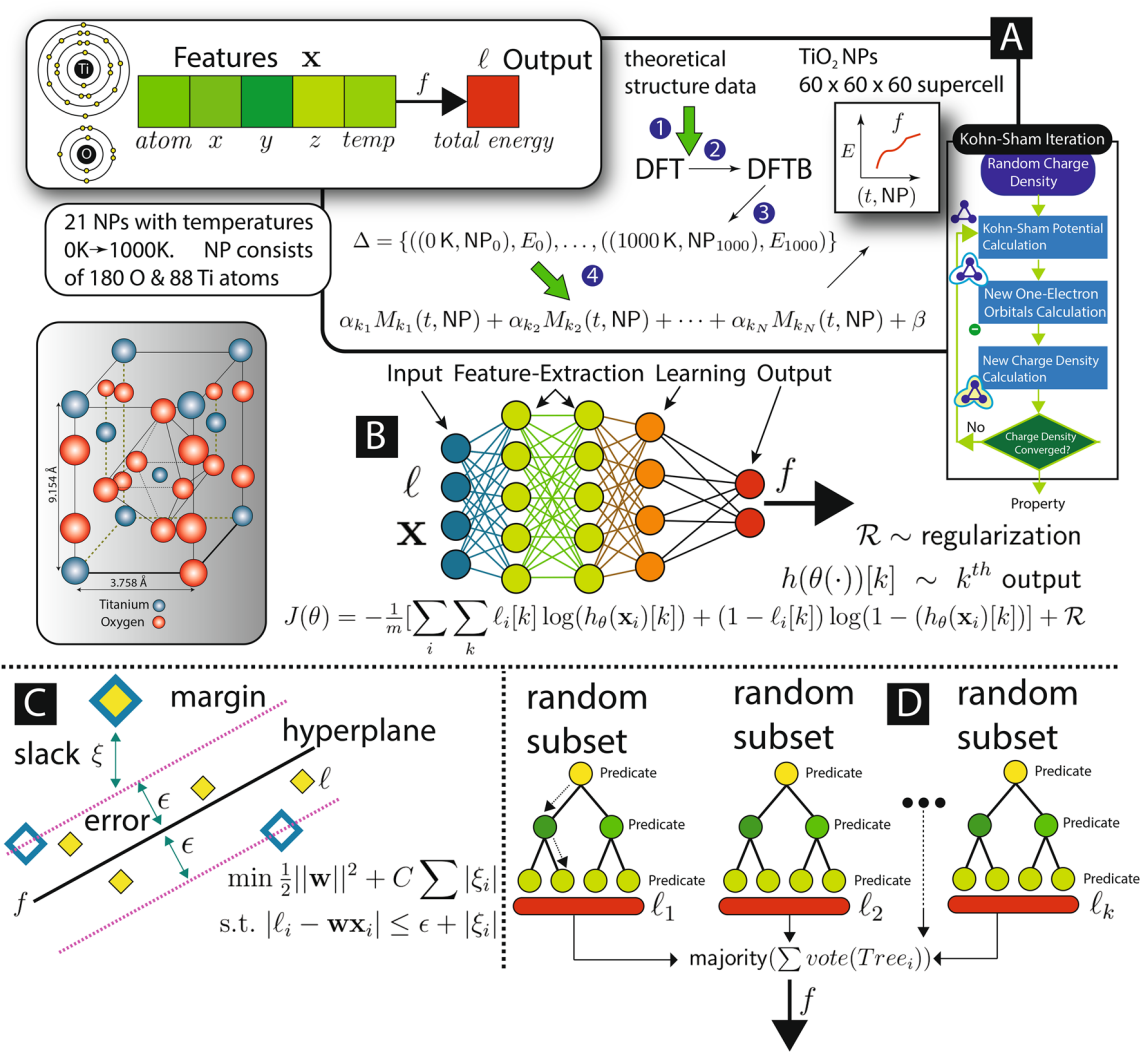
(Inset right) Flowchart of the algorithm used in the self-consistent solution of the Kohn–Sham equations. Cooperative model framework (co-model) that leverages an ensemble of models driven by data. (Top left) are the features and label. We show a sample of the diversity of models: neural nets, support vector machines, random forests. (Top middle) the steps 1–5 using co-modelling for ${\mathrm{TiO}}_{2}$ NPs. Use DFT/DFTB (1–3) to generate minimal viable data (21 data points), select, train, co-model (4–5).

To aid discussion we write $t\in {\mathbb {N}}_0$ for temperature Kelvin and ${\textsf {NP}} = \{((x,y,z),A)|x,y,z \in {\mathbb {R}}\ \wedge \ A \in \{{\mathrm{Ti}},{\mathrm{O}}\}\}$ for a collection of nanoparticle position and atom type. Using DFT/DFTB we computationally determine total Kohn–Sham energy $E_{ij}$ given temperature $t_i$ and nanoparticles ${\textsf {NP}}_j$ (since $i=j$ we use only one subscript). The data set has 21 values:

5 $$\begin{aligned} \Delta= & {} \{((0\,{\mathsf {K}},{\textsf {NP}}_0),E_0), ((50\,{\mathsf {K}},{\textsf {NP}}_{50}),E_{50}), \ldots , ((1000\,{\mathsf {K}},{\textsf {NP}}_{1000}),E_{1000})\} \end{aligned}$$

where $(t,{\textsf {NP}})$ is called the feature set and *E* the label, building a “best” function *f* that, given feature values, gives energy using ML parlance. In this work we are interested in investigating how a linear ensemble of disparate ML models performs. We settled on a linear model, since it is among the simplest, best understood, and most widely used. The types of ML models are quite divers *e.g.*, random forests, support vector machines, neural networks, *k*-nearest neighbor. Detailed discussion of each model is not feasible here, but links to the implementations used in this work are given in the next section. We train every model $M_k$ from a set of candidates using default parameters. Models that either individually perform poorly or are correlated are removed. An linear ensemble of models is further refined yielding *f*

6 $$\begin{aligned} f(t,{\textsf {NP}})\equiv & \,{} \alpha _{k_1}M_{k_1}(t,{\textsf {NP}}) + \alpha _{k_2}M_{k_2}(t,{\textsf {NP}}) + \cdots + \alpha _{k_N}M_{k_N}(t,{\textsf {NP}}) + \beta \end{aligned}$$

where $\alpha , \beta \in {\mathbb {R}}$ are coefficients and constant. Using an additional tuning-parameter grid each candidate model is either optimized for its parameters or removed *via* cross-validation. The simplicity of Eq. (6) belies its power—any value $t \in [0\,{\mathsf {K}},1000\,{\mathsf {K}}]$ and acceptable ${\textsf {NP}}$ for ${\mathrm{TiO}}_{2}$ can be determined directly bypassing the traditional DFT/DFTB technique saving time while preserving fidelity discussed in “Methodology and experimental results” section (Fig. 3).

### The cooperative model framework (co-model)

Presented here is a new approach to predicting Kohn–Sham total energy of nanoparticles by combining a set of single runs of DFT/DFTB with a linear ensemble of disparate ML models we call the cooperative modelling framework (co-modelling)—the results are rapid and accurate. A model’s individual performance criterion considers either (1) Mean Absolute Error (MAE), (2) Root Mean Square Error (RMSE), or (3) *R* Squared (${\mathrm {R}}^2$). Co-modelling consists of five steps:

1. Compute KS energy for a small number of temperature, nanoparticles pairs. We call this data $\Delta$ shown in Eq. (5) and Algo. lines 4–8.
2. Split the data into training data (used to build individual models) and test data (used to assess models quality). We call these data $\Delta _{Train}, \Delta _{Test}$, respectively in Algo. lines 9–10.
3. Build a set of candidate models from a large corpus of models ${\mathscr {M}}$. In this work we are utilizing a possible size of over 230 models. A model is ignored if it is either performing poorly or takes longer than an arbitrary, fixed amount of time to complete ($> 1\,{\mathsf {hr}}$) in Algo. lines 10–16.
4. Of pairs of correlated models remaining, remove the poorer performing in Algo. lines 17–18.
5. Construct a linear ensemble, possibly refining the set of models further, by tuning parameters or pruning returning the function shown in Eq. (6) and Algo. lines 19–21.

The initial model class $|{\mathscr {M}}| = 230$ considers all regression models in caret^61^ which is a popular ML library in R programming language. All models are initially run with default parameter values and become candidates if they complete computation in less than $1\, {\mathsf {hr}}$. To construct the linear ensemble we leverage a popular R package extant in caret called caretEnsemble^62^. This package relies on caretStack that, from a collection of models, either prunes or optimally tunes parameters. Discussed next are descriptions and some characterizations of the gamut of models used. ${\mathscr {M}}$ consists of: lm: Linear regression; glm: Generalized Linear Model; lmStepAIC: Generalized Linear Model with Stepwise Feature Selection, gcvEarth: Multivariate Adaptive Regression Splines, ppr: Projection Pursuit Regression, rf: Random Forest, xgbDart,XgbTree, xgbLinear: Extreme Gradient Boosting; monmlp:Monotone Multi-Layer Perceptron Neural Network; svmLinear, svmRadial: Support Vector Machines (SVMs) with Linear and Radial Kernel Functions; knn: K-Nearest Neighbor; rpart: Decision Tree (cart); gaussprLinear: Gaussian Process; icr: Independent Component Regression. We categorize these ML models as (1) ensemble: RF xgbDart,XgbTree, xgbLinear and (2) and non-ensemble models: lm, glm, icr, lmStepAIC, ppr, gaussprLinear, gvcEarth, svmLinear, knn, rpart, monmlp, svmRadial. Unlike non-ensemble models where only a single model is being built, ensemble models^63^ consist of collections of the same models constructed randomly. More detailed description is provided next.

#### Co-model ensemble models

In RF the ensemble is a collection of DTs constructed with bagging (*i.e.*, independently of each other), whereas XGB builds weak learners (with any type model) in an additive manner—one weak learner at a time. In ensembles, the final decision is given by combining the predictions of all models (“voting”). XGB lets weak learners vote along the way while RF lets all DTs vote at the end after building all trees. Each model is built using a different sample of the training data and, thus, the sampling method is among the factors which determine the final models. Voting strategy is another factor that can change the final prediction, *e.g.*, weighted or unweighted voting where unweighted voting gives an equal weight to the each DT model. RF is quite popular due to its robustness, *e.q.*, remote sensing^64^, land-cover classification^65^, network intrusion detection system^66^, sleep stage identification^67^.

Error correlation among the trees and the strength of the DTs are estimated over the *out of bag* data which is the data remaining from bootstrap sampling. The trade-off between the margin which shows how well a single DT separates a correct class from an incorrect class and the correlations between the trees determines how well RF will perform. Breiman^68^ is the first RF paper and^69^ gives a review of RF algorithm. Differing approaches to improve RF; weighted voting and dynamic data reduction^70^, through sampling^71^, improving data^72^, with clustering^73^.

The stochastic gradient algorithm^74^ improves^75^, the first gradient boosting algorithms for big data. Extreme Gradient Boosting (XGBoost/XGB)^76^ is a scalable gradient tree boosting algorithm proven to work well in many areas, *e.g.*, finance^77^, bioinformatics^78^, energy^79^, music^80^. Unlike RF, the cost function given in Eq. (7) is solved in an additive manner since it is not possible to optimize it in Euclidean space using the traditional optimization methods; it can be solved using second-approximation^81^.

7 $$\begin{aligned} J^t= \displaystyle \sum _{i=1}^{n} h(y_i,\hat{y_i}^{t-1} + f_t({\mathbf {x}}_{\mathbf{i}})) +\omega (f_t) \end{aligned}$$

where $\hat{y_i}$ represents the prediction for the $i^{th}$ data point and

$$\begin{aligned} \hat{y_i}= \sum _{k=1}^{K} f_{k}({\mathbf {x}}_{\mathbf {i}}), f_{k}\in {\mathscr {F}} \end{aligned}$$

${\mathscr {F}} = \{ f({\mathbf {x}}) = g_{q({\mathbf {x}})}\} (q; {\mathbb {R}}^m \rightarrow T, g\in {\mathbb {R}}^T)$ represents the trees’ space. *K*; additive functions’ number, ${\mathbf {x}}_{\mathbf {i}}$; $i^{th}$ data point, *n*;  data size, *m*;  input variables’ number, *t*;  iteration number. *q* is the structure of the trees and $f_k$ is an output of an independent tree structure *q* with a leaf weight. *h* denotes a differentiable convex loss function and measures the difference between the true model *y* and the predicted model ${\hat{y}}$. $\omega$ is the penalization parameter which is used to tune the complexity of the model and avoid the overfitting problem.

#### Co-model non-ensemble models

In this section we briefly explain non-ensemble models under two categories: (1) linear: lm^82^, glm^83^, icr^84^, lmStepAIC^85^, ppr^86^, gvcEarth^87,88^, svmLinear^89^ and (2) non-linear: knn^90^, rpart^91^, gaussprLinear^92^, monmlp^93^, svmRadial^89^. Since the models under the same category learn the data according to different strategies, the final models they create are different from each other. For example, although lm can only learn linear relationships in the data, gvcEartch can learn non-linear relationships as well. icr decomposes training data into linear combinations of components which are independent of each other as much as possible. gvcEarth partitions input data into piece-wise linear segments with differing gradients. Linear segments are connected to obtain basis functions which are used to model linear and non-linear behaviors. svmLinear treats the “best” line problem as the maximal margin problem and solves the 2-norm cost functions (least squares error). svmRadial uses radial basis function as kernel function.

lm is Ordinary Least Squares (OLS) under the assumption that residuals distribution is Gaussian. glm searches for the best line by assuming that the distribution of residuals comes from an exponential family klike a Poisson. lmStepAIC, a type of glm, selects the input variables using an automated algorithm. In ppr in an iterative manner the regression surface is modelled as a sum of general linear combinations of smooth functions. This makes ppr more general than stepwise regression procedures. icr decomposes training data into linear combinations of components which are independent of each other as much as possible. gvcEarth partitions input data into piecewise linear segments with differing gradients. Linear segments are connected to obtain basis functions which are used to model linear and non-linear behaviors. gaussprLinear is a probabilistic regression model and a probability distribution over linear functions that fits training data. rpart is a single tree model trained by selecting an optimal attribute (feature) then partitioning data based on the class data for each value in the active domain. This is akin to Quinlan’s C4.5. The optimal attribute is chosen using a variety of metrics such as Gini or information gain where each metric can lead to produce a different DT^91,94–96^. Subsequently the training data points are sorted to the leaf nodes. The algorithm runs until the training data points are classified to an acceptable threshold; otherwise it iterates over new leaf nodes. After building DTs are pruned to prevent overfitting (performing well on the training data, but poorly over test data). Pruning greatly impacts performance^95^. A neural network is a weighted, directed graph where nodes input/output and edges weights. monmlp is a recurrent network with a monotone constraint which is used to monotonically increase the behavior of model output with respect to covariates. monmlp handles overfitting using bootstrap aggregation with early stopping. In knn, the data itself is used to make a prediction (*lazy learning*). knn first searches for *k*-similar objects in the training data with some distance metric and then uses voting. The choice of *k* can drastically change the model’s prediction.

## Methodology and experimental results

In this section we first explain how we generate the minimal viable data and then present our novel machine learning framework over the ${\mathrm{TiO}}_{2}$ NPs and share our findings.

### Data set: ${\mathrm{TiO}}_{2}$ nanoparticles

**Figure 4:**
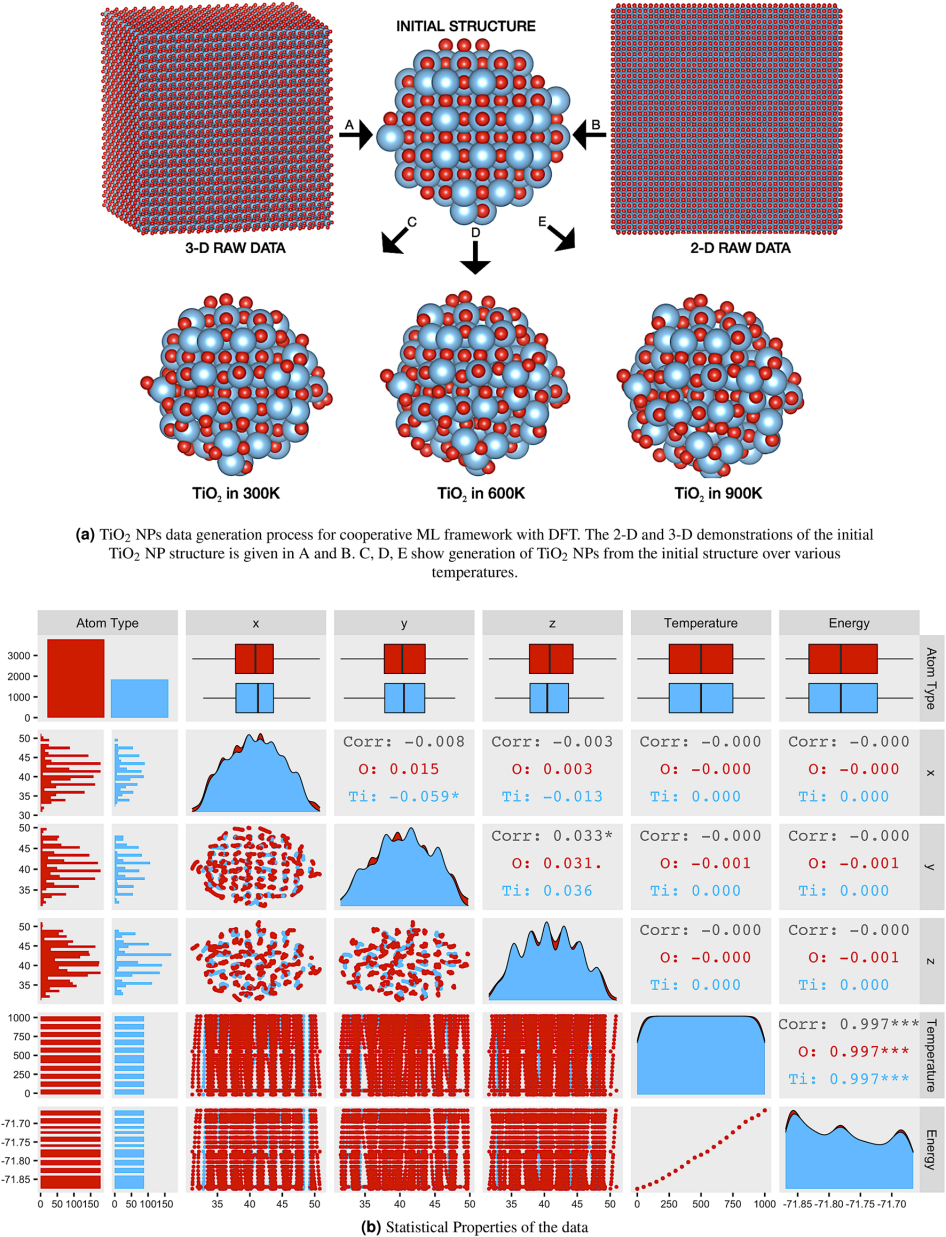
The summary of statistical properties of training data. The abscissa and ordinate show the variable values. Red and blue colors represent O and Ti atoms, respectively. Each input feature and bivariate relationships between the input variables are visualized depending on Ti and O atoms.

In Fig. 4 illustrates the theoretical supercell data (3-D, 2-D raw data) of ${\mathrm{TiO}}_{2}$ NPs and some statistical properties of the portion of training data. The process begins by carving ${\mathrm{TiO}}_{2}$ NPs from a bulk 60 $\times$ 60 $\times$ 60 supercell. Next, structures at different temperatures to attain various ${\mathrm{TiO}}_{2}$ NPs are computed. Figure 4a illustrates three different NPs generated at $300\, {\mathsf {K}}$, $600\, {\mathsf {K}}$, and $900\, {\mathsf {K}}$. Structural data is taken Kurban et al.^97^ with a detailed description about the initial geometry of the ${\mathrm{TiO}}_{2}$ NP model. The structural, electronic and optical properties of twenty-one ${\mathrm{TiO}}_{2}$ NP models were obtained from the density functional tight-binding (DFTB) calculation^98–100^ at different temperatures $[0\,{\mathsf {K}},1000\,{\mathsf {K}}]$. Each NP consists of 180 O and 88 Ti atoms. In Fig.  4b shows statistical properties of ${\mathrm{TiO}}_{2}$ NPs used in this study, *e.g.*, distribution, correlation. The input variables are *atom*, three-dimensional geometric locations of Ti and O atoms (*x*, *y*, *z*) and *temperature*. The *Energy* variable is the output variable. We observe that the variables are linearly non-correlated.

### Using co-modelling to predict energetic properties

Let ${\mathsf {AC}}, {\mathsf {TE}}$ stand for an initial atomic configuration and Kohn–Sham total energy. Treating *DFTB* as a function, total energy is a function solely of atomic configuration shown as computation (8):

8 $$\begin{aligned} DFTB({\mathsf {AC}})\rightarrow & {} {\mathsf {TE}} \end{aligned}$$

Taking temperature into account, we compute the triple (9):

9 $$\begin{aligned} DFTB({\mathsf {AC}}^{\mathsf {i}},t)\rightarrow & {} ({\mathsf {AC}}^{{\mathsf {i}},t},{\mathsf {TE}}^t,t) \end{aligned}$$

where ${\mathsf {AC}}^{{\mathsf {i}},t}$ is the new atomic configuration using an initial configuration ${\mathsf {AC}}^{\mathsf {i}}$ paired with total energy at temperature *t*. The co-model is built from 21 different values described in computation (9).

Our initial examination of the co-model itself might lead one to believe that temperature is the principle driver of total energy. This is not the case. The co-model is learning the *relationship of the triple* not simply temperature as a predictor of total energy. Our results show (including an active web service) that the *triple* is so well characterized that, given ${\mathsf {AC}}^{\mathsf {i}}, {\mathsf {AC}}^{{\mathsf {i}},0}, {\mathsf {AC}}^{{\mathsf {i}},50},\ldots , {\mathsf {AC}}^{{\mathsf {i}},1000}$ and temperature $t \in [0\,{\mathsf {K}},1000\,{\mathsf {K}}]$, it can accurately predict ${\mathsf {TE}}^t$. The mechanics of the computation are not easy to describe. Underlying most of AI is the recurring conundrum: the model performs well, but how it works is often impossible to explain. Indeed, an active area of AI is explainability^101–103^. Interestingly, work is now taking place that allows AI to completely describe the phenomenon^104^. At this point, the results show an effective technique to drastically reduce run-time. It remains for future work how, if even possible, we can make some human-interpretable sense of the computation.

Figure 3(top middle) shows the general stops to constructing a co-model that can efficiently, quickly, and effectively predict structural, electronic and optical properties of NPs. The framework starts with generating a minimal viable theoretical data set using DFT/DFTB (steps 1–3). The data is randomly partitioned (step 4) into training and test boosting ($| \Delta _{Training}| > |\Delta _{Test}|)$. Cross-validation is a standard technique to assess the quality of models. Pearson’s correlation is measured among the model cohort allowing elimination of models that are either highly correlated with a better model or perform badly. The final co-model is built and quality measured (step 5). A more detail description of these steps are provided next. In this work, total energy is eV/atom.

### The application of co-modelling to anatase ${\mathrm{TiO}}_{2}$ nanoparticles

**Figure 5:**
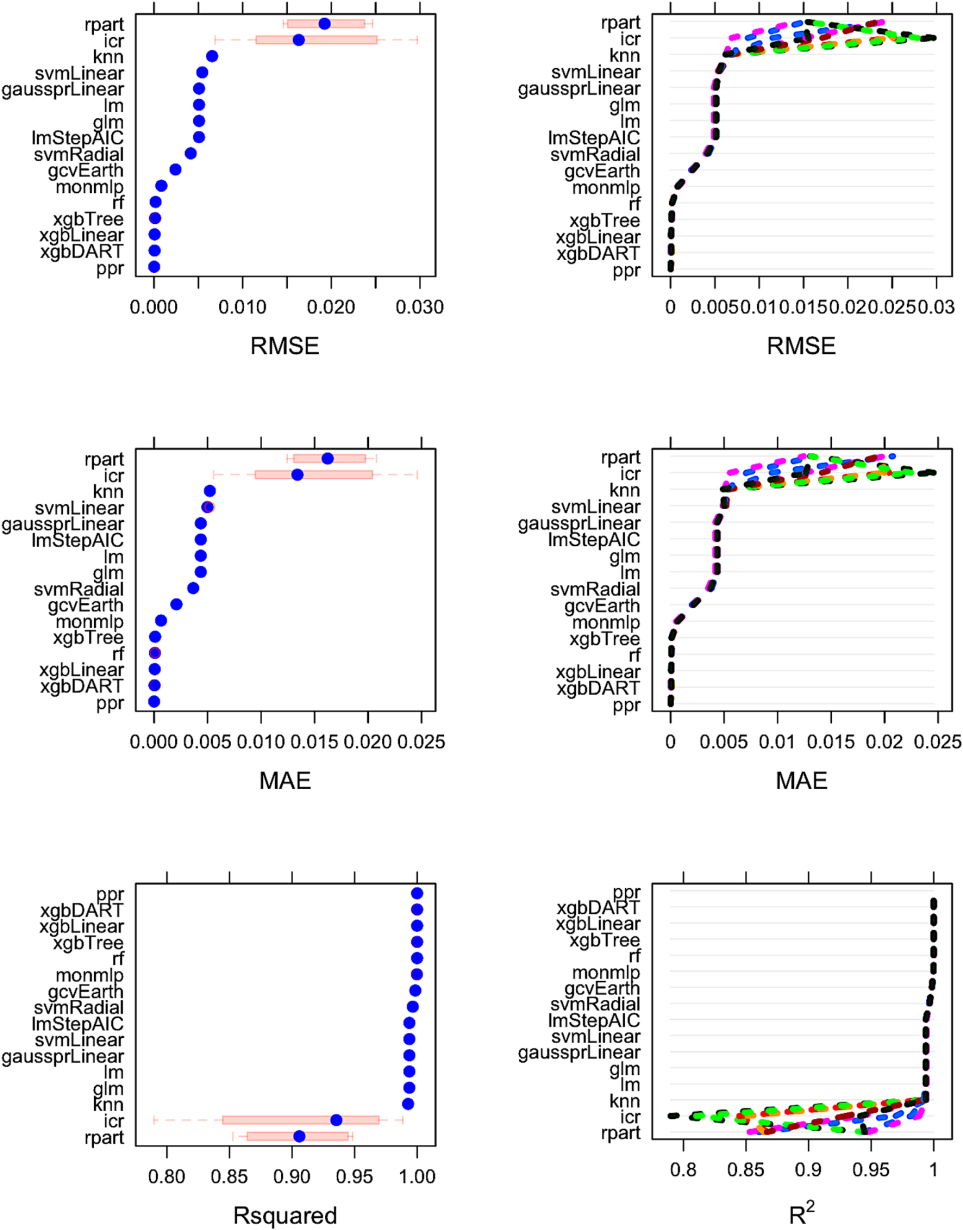
Observing RMSE, MAE, R$^2$ of each model constructed over the training data using Plot Vertical Box-and-Whisker plots. The tenfold cross-validation results of each model using various metrics are shown in this figure. icr and rpart tend to build models with high variance. Considering the experimental results, the best performing ML algorithm on the training data is ppr. rpart has the worst MAE, RMSE and ${\mathrm {R}}^2$ values.

Referring to Algo. 1 experimental detail is presented. The construction begins with 21 ${\mathrm{TiO}}_{2}$ NPs at temperatures ranging over $[0\,{\mathsf {K}},1000\,{\mathsf {K}}]$ from DFT/DFTB. Training data, $\Delta _{Training}$, represents 75% of original data and test data, $\Delta _{Test}$, the remaining 25%. The ratio Ti/O are the same in both $\Delta _{Training}$ and $\Delta _{Test}$. In DFTB, the symmetries of the studied NP models were broken under heat treatment; thus, the co-model ensemble and other ML models were trained over non-symmetric NPs^105,106^. The goodness of the models use three metrics that produce values in [0, 1]: (1) Mean Absolute Error (MAE), (2) Root Mean Square Error (RMSE), (2) R Squared (R$^2$). Metrics (1) and (2) are error metrics; thus lower values are better. MAE and RMSE are distance metrics that, as the model improves, describe the *deviation* as a magnitude from the actual data (DFT/DFTB) and not the actual accuracy of the model. R$^2$, in a regression model, represents the proportion of variance in the input variables (dependent) and that can be explained by the output variable (independent). The higher R$^2$, the better model is. tenfold cross-validation is used to assess the quality of the models and the summary is given in Fig. 5.

**Figure 6:**
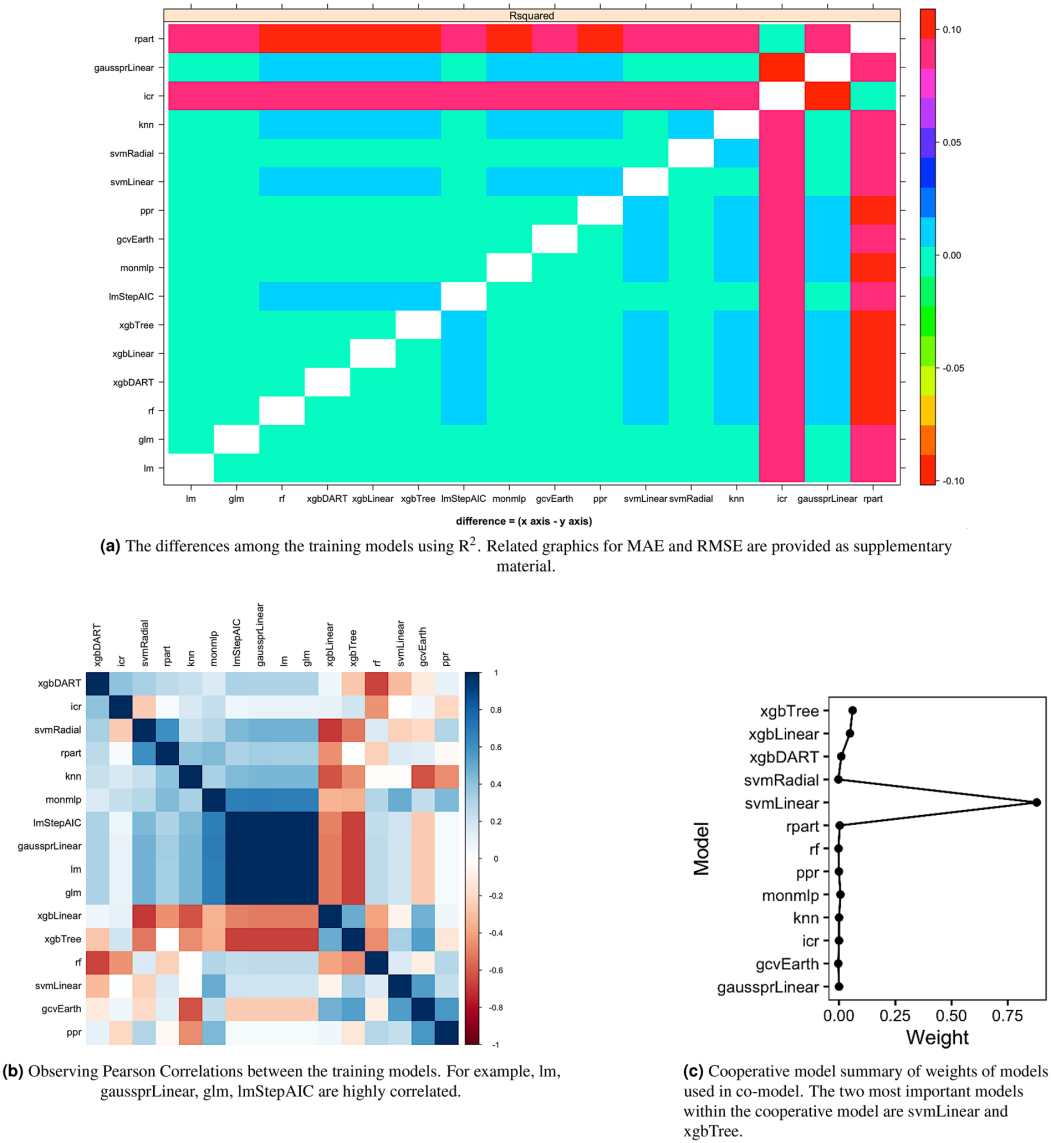
Determining the models for the cooperative model (**a**, **b**) and the details of the cooperative model (**c**).

**Figure 7:**
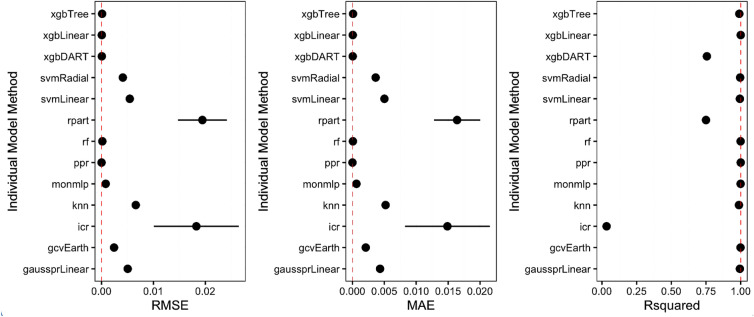
The cooperative model *versus* the classical ML algorithms: comparison of the cooperative model with the classical ML models over the training data set. The red-dashed line represents the cooperative model. We observe that the cooperative model’s performance is even slightly better the most accurate classical ML models.

The best performing traditional ML model is ppr in the training step. The experimental results indicate that the performances of XGBoost algorithms (xgbDart, xgbLinear, xgbTree) and rf are similar to ppr. Although some single ML models such as ppr, xqbDart, xqbLinear, perform well on training and test data, the our framework makes it possible to create more accurate models than any of these single ML models. Any new data may not be well-modelled by a single ML model; the co-model will always perform well. Figure 6 highlights pairwise training model differences: Fig. 6a shows ${\mathrm {R}}^2$; Fig. 6b shows linear correlations; Fig. 6c shows a Pearson heat map. Figures for MAE and RMSE are provided as the supplementary material for space reasons. After finding the best models among various regression models, correlations among the models, created during the training step, were measured. The results show that lm-lmStepAIC, lm-gaussprLinear, glm-lm, glm-lmStepAIC,glm-gaussprLinear pairs are highly correlated. We only use gaussprLinear, since its performance is better than others over the training data. The co-model is constructed using the remainder of the cohort. Note that there was none that performed poorly over $\Delta _{Training}$. The performance comparison of traditional ML models and cooperative model over the training data is presented in Fig. 7. The red-dashed line represents the cooperative model and we observe that our cooperative model performs better than the rest of the ML models. For example, over the training data, the RMSE was $1.5 \times 10^{-10}$ where ppr, the best traditional model, had $1.6 \times 10^{-10}$ RMSE. Figure 6c demonstrates the relative importance of individual models in the cooperative model. We observe that svmLinear, xgbTree, xgbLinear and xgbDART are the most important models, respectively, in the cooperative model, and svmLinear is the most dominant one. Finally, we present the performance of the cooperative model and other models over the testing data in Table 1. Based on all the metrics the results demonstrate that co-modelling performed superior than all other traditional single models. Our best model is embedded to the web and can be easily tested (https://hasan.shinyapps.io/app_1/).

**Table 1:** Performance comparison of co-modelling and classical ML models over ${\mathrm{TiO}}_{2}$ test data.

Model cooperative	RMSE	MAE	R$^2$
	$1.9\times 10^{-5}$	$7.2\times 10^{-6}$	1.00
ppr	$2.2\times 10^{-5}$	$7.4\times 10^{-6}$	1.00
xqbLinear	$6.6\times 10^{-5}$	$5.8\times 10^{-5}$	1.00
xqbDart	$9.7\times 10^{-5}$	$8.4\times 10^{-5}$	1.00
rf	0.0001	$8.4\times 10^{-5}$	1.00
xgbTree	0.0001	$9.8\times 10^{-5}$	1.00
monmlp	0.0008	0.0006	1.00
gvcEarth	0.002	0.002	1.00
lmStepAIC	0.004	0.0042	0.99
glm	0.004	0.004	0.99
lm	0.004	0.004	0.99
gaussprLinear	0.004	0.004	0.99
svmRadial	0.005	0.003	1.00
svmLinear	0.005	0.005	0.99
knn	0.006	0.004	0.99
icr	0.006	0.005	0.99
rpart	0.022	0.018	0.88

## Summary and conclusion

We have demonstrated that by pairing DFT/DFTB with a novel, data-driven ML framework—and surprisingly a modicum of data of 21 NPs—we can bypass traditional run-time bottlenecks scientists face when using DFT/DFTB alone. Co-modelling builds a linear ensemble of models that accurately predicts the structural, electronic, and optical properties of nanoparticles (NPs). The collective time to build the ML portion is relatively minuscule and can even be done *ab initio*. Our solution is open-source and only requires a standard hardware—a laptop. We focused on ${\mathrm{TiO}}_{2}$ NPs to predict the Kohn–Sham (KS) total energy given some temperatures. The diversity of the ensemble is striking: Extreme Gradient Boosting (xgbDart, xgbLinear, xgbTree), Support Vector Machines (svmLinear, svmRadial), Neural Network (monmlp), Random Forest(rf), Linear and Non-linear Regression (gaussprLinear, gcvEarth, ppr, icr), Decision Tree (rpart), K-nearest Neighbor (knn) algorithms. These results are quite promising and the cooperative model has 1 of R$^2$ value and almost 0 of Root Mean Squared error (RMSE) and mean absolute error (MAE) over the training and test data. Data continually changes—new, improving, conjectured. The data-driven co-model technique is well-suited in these situations. Future work includes determining what the best and minimal data can be and ensemble, whether instances of this model differ for materials or types of properties predicted, generating data for general use. Additional future work includes elucidating how the co-model is understanding the relationship possibly leading to a different, perhaps simpler, description of atomic configuration, temperature, and total energy. Another intriguing path is to reverse the computation—can we determine the most likely atomic configuration or class of configurations that would give rise to the total energy. Examining other metal nanoparticles must be investigated to ensure this result is not simply peculiar to ${\mathrm{TiO}}_{2}$. We are also interested in studying extreme temperatures where experimentation is difficult at best. Finally, code and data are publicly accessible and the cooperative model is available on the web.

## Supplementary Information

Supplementary Information.

## Data Availability

The raw/processed data required to reproduce these findings can be shared if requested. For questions regarding data, contact Hasan Kurban at hasan.kurban@sjsu.edu.
