# Supplementary material for: Rapidly predicting Kohn–Sham total energy using data-centric AI
Source: Sci Rep. 2022 Aug 24;12:14403. doi: 10.1038/s41598-022-18366-7 (PMC9402589; doi:10.1038/s41598-022-18366-7)
Supplement: Supplementary file 1 — Supplementary Information. [file 41598_2022_18366_MOESM1_ESM.pdf]

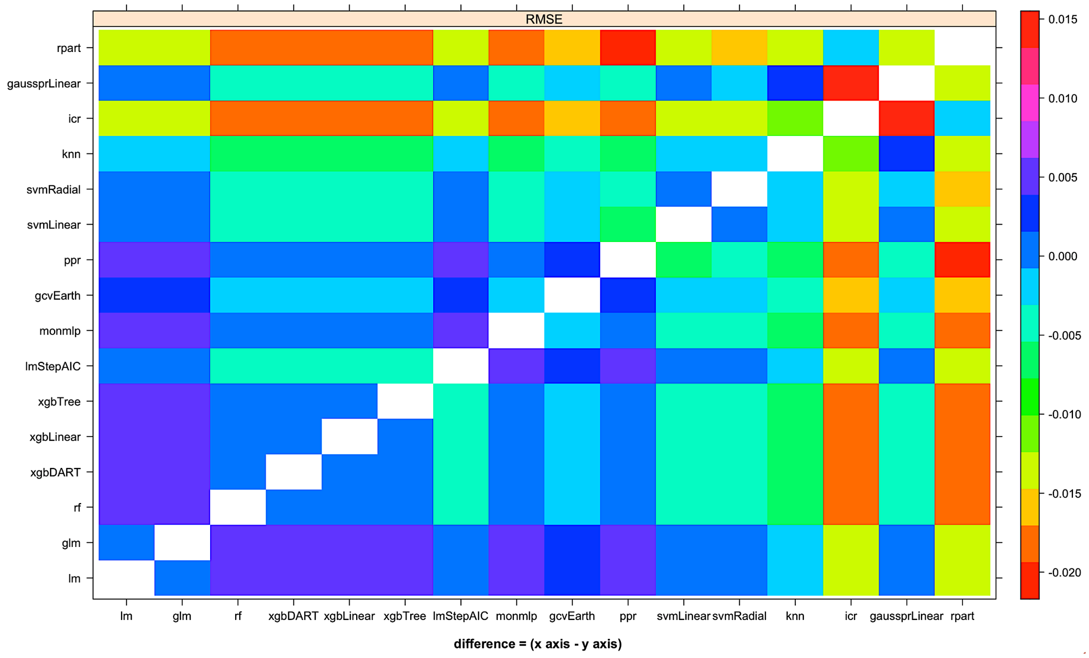

(a) Observing the differences between the training models using RMSE.

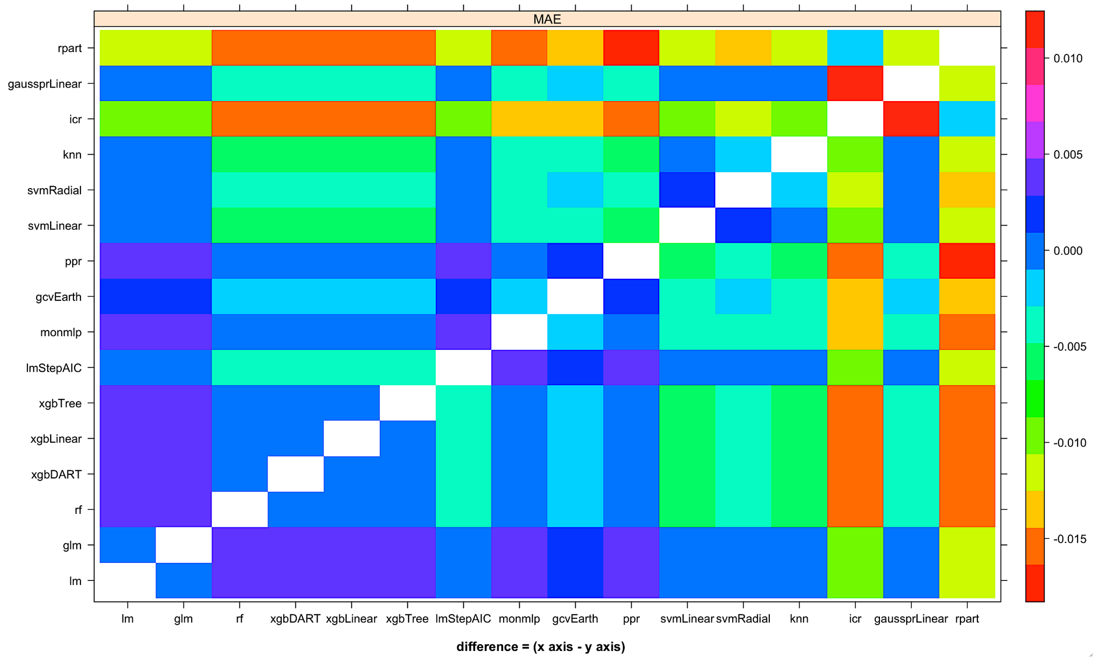

(b) Observing the differences between the training models using MAE.

Figure S1. Selection of classic ML models, which are important for the cooperative model, using different metrics.

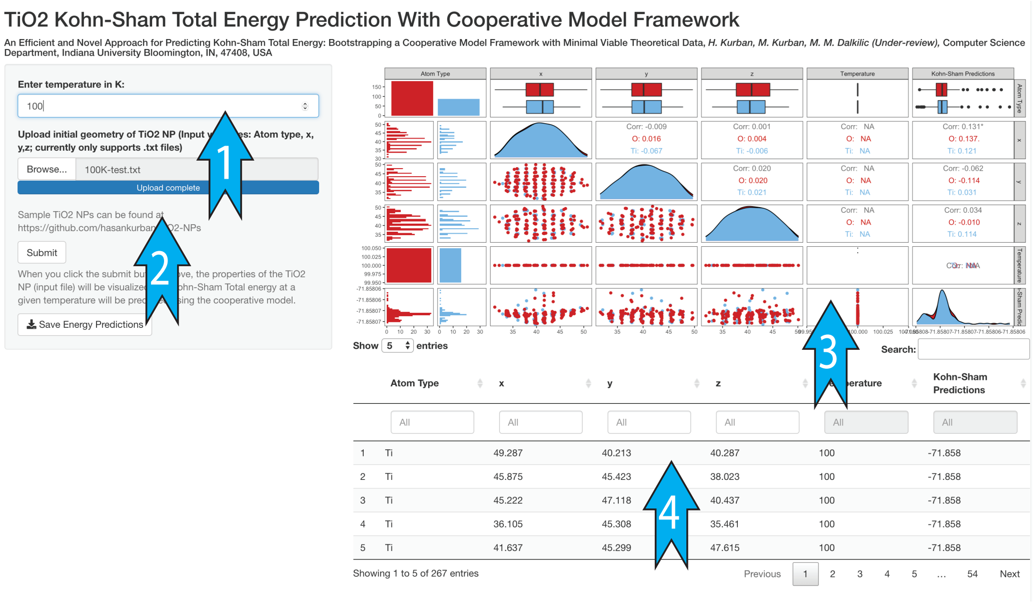

Figure S2. Web service for TiO_2 NPs. The inputs are (arrow 1) temperature and (arrow 2) atom geometries. The Khon-Sham total energy prediction (arrow 4) and statistical properties of data (arrow 4) are returned. The service is using the co-model without having to revisit DFT/DFTB.
